# Hypertensive Crisis in Pregnancy with COVID19: Confirmed with rt-PCR for Nasopharyngeal Swab

**DOI:** 10.1155/2020/8868952

**Published:** 2020-11-27

**Authors:** Sajith Bandara, Anura Ruwanpathirana, Dhammika Nagodawithana, Samantha Alwis

**Affiliations:** De Soysa Hospital for Women, Colombo 10, Sri Lanka

## Abstract

The novel coronavirus has already spread across the geographical borders to 213 countries and self-governing territories. However, the effect of SARS-CoV-2 infection on pregnant mothers is still poorly understood and sparsely documented. Here, we present a case of a primi mother, who presented with diarrheal episode and proceeded to a hypertensive crisis and placental abruption with rt-PCR (nasopharyngeal swab) confirmed for COVID19. SARS-CoV-2 enters and downregulates host cell-bounded enzyme ACE2 (angiotensin-converting enzyme). This activates the renin angiotensin aldosterone mechanism (RAAM). The activation of RAAM plays a pivotal role in the pathophysiology of hypertensive emergencies. Hence, there is a theoretical possibility of hypertensive crisis associated with ACE2/RAAM dysfunction in pregnant mothers who have COVID19. Therefore, close monitoring of blood pressure and early intervention are of paramount importance in anticipating and preventing serious complications related to hypertension in pregnancy in mothers who have tested positive for SARS-CoV-2, especially in this pandemic situation. Emergency hospital admission and urgent care must be afforded to mothers presenting with high blood pressure with the features suggestive of COVID19 as they are at a risk of rapid deterioration.

## 1. Introduction

The novel coronavirus was first documented in December 2019, in Wuhan, a city in the Hubei Province of China, due to clusters of pneumonia associated with severe acute respiratory syndrome [1]. The clinical course of the disease ranges from mild to critical illness. 20% of cases remain asymptomatic throughout the course [2]. Majority of the documented cases have mild symptoms while 5% progress into critical disease with complications. The overall case fatality rate is approximately 2.1% among confirmed cases [3]. Apart from respiratory and cardiovascular symptoms, some patients experience gastrointestinal disturbances such as nausea, vomiting, and diarrhea as the first symptom [4, 5]. Diarrhea was one of the common symptoms seen in 7% among 118 pregnant mothers in a case series observed in China [6]. However, there was no significant increase in the incidence of intrauterine fetal death, neonatal death, or early trimester loss among pregnant mothers as per the stipulated study.

## 2. Case Report

A 19-year-old previously normotensive (up to 30-week follow-up; her blood pressure measurements were normal, but she defaulted her last clinic visit due to lockdown) primi mother presented from an area in Colombo, Sri Lanka, which was quarantined due to the rapid spread of COVID19 among its residents. She presented with reduced fetal movement for 6 hours and diarrhea 5 times a day for the last 24 hours. She was 33 weeks pregnant, and the uterine size was compatible with her period of gestation. Thus far, her pregnancy was uncomplicated with no significant medical or surgical problems. She had no history of allergies. On admission, her blood pressure was 170/100 mmHg with protein 3+ in urine dipstick analysis. She did not have symptoms or signs of impending eclampsia other than upper abdominal mild pain and tenderness.

Urgent sonography revealed an intrauterine fetal demise and placental abruption. Fetal growth was compatible with 31 + 3/7 weeks without features suggestive of growth restriction. The amniotic fluid index was normal. Haematology revealed Hb of 5.2 g/dl (NRBC: 0.7%), WBC of 24.38 (Net: 81.7%, Lym: 13.2%), and Plt: 210; other investigations including clotting, renal, and liver function tests were found to be normal. Her blood pressure was controlled with 2 bolus doses of Hydralazine 5 mg 30 minutes apart. Blood transfusion was started. Once the patient stabilized, an emergency caesarian section was performed with a full personal protective gear. There was a significant amount of free fluid noted within the abdominal cavity; Couvelaire uterus was noted. The fetus showed signs of recent demise with fetal weight appropriate for gestational age. A large blood clot was noted separating the placenta confirming the ultrasound diagnosis of abruption. Later, postpartum bleeding ensued which was managed with a Bakri® balloon tamponade device. After 3 consecutive blood transfusions, her Hb level was 8.4 g/dl. The biophysical parameters remained stable throughout the immediate recovery period. Her rt-PCR for SARS-CoV-2 from nasopharyngeal swab was tested to be positive on the following day.

## 3. Discussion

The novel coronavirus is a RNA virus. Its genome and structure closely resemble the SARS-CoV while the main animal vector is thought to be a bat. A membrane S glycoprotein has been demonstrated which closely resembles the protein found in the bat coronavirus except for a single amino acid [7]. This adherence molecule facilitates the endosomal internalization of the virus through the host cell-bounded enzyme ACE2 (angiotensin-converting enzyme) [8, 9]. Initial infection through the respiratory tract is thought to be via type 2 pneumocytes in the lungs [10]. However, the virus may affect other organ systems depending on the level of expression of ACE2 on their cell membrane [11, 12]. The gastrointestinal tract, brain, myocardium, and kidney abundantly express the ACE2. Moreover, the effects of SARS-CoV-2 are believed to be local hyperinflammation of each system [13]. There is evidence to state that the activation of RAAM (renin angiotensin aldosterone mechanism) and downregulation of ACE2 significantly contribute to the pathological process of lung injury in SARS-CoV [14]. ACE2 is a new component of RAAM, which was identified in 2000. It hydrolyses angiotensin 2 to angiotensin 1-7 [15]. In animal studies, overexpression of ACE2 significantly reduces the baseline blood pressure in chronically hypertensive mice [16].

The ACE2 enzyme and the rest of the components of uteroplacental RAAM play a key role in maintaining blood pressure, electrolyte, and water homeostasis in a pregnant mother [17]. During pregnancy, ACE2 is expressed abundantly in uterus (endometrium and myometrium), placenta, membranes, and fetal tissues. Hence, the transplacental transmission of SARS-CoV-2 is theoretically possible. However, recent studies do not support vertical transmission [18].

In the course of a normal pregnancy, there would be a noticeable increase in plasma angiotensinogen due to the abundancy of oestriol (E3). This would result in excess RAAM activity and facilitate physiological hypervolemia in pregnancy [17]. There is a notable progressive dysfunction in RAAM activity from early placentation among mothers with gestational hypertension and preeclampsia [17]. Furthermore, activation of RAAM plays a pivotal role in the pathophysiology of hypertensive emergencies [19]. Hence, there is a theoretical possibility of hypertensive crisis associated with SARS-CoV-2-associated ACE2/RAAM dysfunction in pregnant mothers. This may be complicated with sudden placental abruption and intrauterine demises.

In conclusion, available clinical information regarding COVID19 effect on gestational hypertension and impact on placental function remains unknown. Whether SARS-CoV-2 precipitates placental dysfunction, adversely affects the blood pressure homeostasis, and causes placental abruption is still unclear. However, there is a theoretical possibility of hypertensive emergencies that may follow COVID19. Therefore, it is important to be vigilant on blood pressure control in pregnant mothers, especially those who already have hypertensive diseases. Emergency hospital admission and urgent attention must be given to mothers presenting with high blood pressure with the features suggestive of COVID19 during the SARS-CoV-2 pandemic as their condition may deteriorate rapidly.

## Data Availability

All the information/data has been attached as references.
